# Mapping and Assessing Existing Digital Skills Training Programs for Health Care Professionals and Health Managers in Europe: Expert-Based Survey for Investigating the Landscape, Accessibility, and Effectiveness of Training Initiatives

**DOI:** 10.2196/71657

**Published:** 2025-08-29

**Authors:** Dimitrios Protogiros, Theologia Tsitsi, Constantina Cloconi, Iolie Nicolaidou, Efthyvoulos Kyriacou, Norbert Couespel, Deborah Moreno-Alonso, Carme Carrión, Ana Claveria, Andreas Charalambous

**Affiliations:** 1 Department of Nursing, Cyprus University of Technology, 30 Archbishop Kyprianos Street, Limassol, 3036, Cyprus, 30 6976531734; 2 Department of Communication and Internet Studies, Cyprus University of Technology, Cyprus, Limassol, Cyprus; 3 Department of Electrical, Computer Engineering and Informatics, Cyprus University of Technology, Limassol, Cyprus; 4 European CanCer Organisation, Brussels, Belgium; 5 Catalan Institute of Oncology, Barcelona, Spain; 6 eHealth Lab Research group, eHealth Center, Universitat Oberta de Catalunya, Barcelona, Spain; 7 Galicia Sur Health Research Institute, SERGAS-UVIGO, Vigo, Spain

**Keywords:** digital training, health, digital skills, digital competencies, mapping

## Abstract

**Background:**

Digital skills training in health is crucial to ensure that the health care workforce is equipped to leverage the potential of digital technologies in delivering efficient and effective care. Identifying existing training programs can be valuable to describe gaps and opportunities for acceleration in the digital age.

**Objective:**

This study aimed to map and assess existing continuous education and professional development training options in digital skills in health.

**Methods:**

As part of the EU-funded project entitled “TRANSiTION - Digital Transition and Digital Resilience in Oncology,” an expert-based approach was implemented to identify training programs in 14 European countries. The data were collected via an online survey that was developed for the purpose of this study and consisted of 23 questions categorized in 5 domains: general information, reaction, learning, behavior, and results. The analysis was performed using the 4-level Kirkpatrick model and the Digital Competence Framework for Citizens.

**Results:**

The analysis of the data showed that 39.6% (19/48) of cases reported no official training in digital skills for the health care workforce, despite the fact that in 95.8% (45/47) of the cases digital solutions were used in the daily practice. Digital skills were a professional qualification in 31.3% (4.38/14) of the countries, and 32 out of 57 programs were provided by academic institutions. Half of the countries scored lower than the mean in the overall performance status according to the Kirkpatrick model, which reflects the gap in knowledge and skills of the workforce. Countries scored at the average or lower in all models’ domains: reaction 92.9% (13/14), behavior 78.6% (11/14), learning 71.4% (10/14), and results 50% (7/14). The quality of the programs was poor, as less than half of the competencies were met, and the evaluation reflects the great need for improving health workforce education in digital skills and the application of health technologies in practice.

**Conclusions:**

There was variance in the availability and quality of digital skills training across Europe. The development of a comprehensive training program targeted to improve health care professionals’ and health managers’ knowledge and skills, as well as the incorporation of digital tools into practice, is crucial.

## Introduction

Health care systems in Europe, as in other parts of the world, are being impacted and reshaped by digital transformation [1]. The implementation of digital innovations in health includes the usage of information and communication technologies in health devices and services for improved access to health care and quality of care, as well as increasing health sector efficiency [2]. Another emerging area where digital solutions have shown great promise is that of reducing health inequalities [3]. However, at the same time, they risk exacerbating inequalities by disproportionately benefiting a subset of the population [4].

It is estimated that in the near future, more than 9 out of 10 professions in Europe will require digital skills, recognizing them as one of the most in-demand skills [5]. However, there are significant differences between European Member States in people’s knowledge and ability to use digital solutions, with up to 40% of Europeans lacking basic digital skills [6].

Although evidence supports that the application of digital technologies in health has great potential to improve the accessibility, quality, and flexibility of health care [1,7] and can lead to more equitable and affordable systems by reducing disparities [7], the health care workforce in Europe is lacking in digital skills, which impacts the adoption of technological solutions in daily practice [8]. The successful digitalization of health encompasses the changes to the fundamental principles and approaches of health care professionals’ (HCPs) and nonclinical professionals’ (health managers’) education [1] and requires incentives, organizational, and academic support [9].

A variety of barriers to use digital interventions by the health workforce have been identified and include infrastructure and technical issues, lack of educational resources, psychological barriers, and workload-related concerns [10]. On the other hand, the available evidence suggests that an effective change can be made by integrating digital skills learning into the health science education curriculum and practice and considering the pedagogical design, learning styles, and HCPs’ expectations, and by including institutional elements such as flexibility, access, and costs for continuing education [10,11].

It is becoming clear that in the digital era, there is an urgent need in Europe to upskill and reskill the European health workforce [12] in modern practices to enable the acquisition of digital skills and competencies for current and future needs in prevention, diagnosis, treatment, monitoring, and management of health-related issues and to monitor and manage lifestyle habits that impact health. The first step toward developing targeted strategies and interventions to enhance digital skills and ensure that the health care workforce is equipped to meet the demands of modern health care delivery is the mapping of the current situation in training and education. While previous studies have typically focused on isolated or region-specific digital health training initiatives, often concentrating on select professional groups, the current research addresses a critical gap by systematically mapping and assessing a broad range of digital skills training programs for both HCPs and health managers across Europe, thereby offering a comprehensive and comparative perspective that informs policy development and best practices in digital health care. The aim of this study was to assess the availability and quality of digital skills training programs and educational resources in 14 European countries. It was meant to provide a comprehensive overview of HCPs’ and health managers’ training in digital skills and to help identify gaps, opportunities, and areas for improvement.

## Methods

### Overview

An expert-based approach was implemented for identifying existing programs for digital skills education and training for HCPs and health managers in 14 European countries. The above strategy was used because information on the specific topic was not available in the literature, and when some of the information was available, it was fragmented and beyond the scope of this study. This method also provides nuanced, context-specific insights that broader survey or literature review methods might overlook, especially in a complex and evolving area such as digital skills training. Experts can capture regional variability and emerging best practices, ensuring that the mapping and assessment reflect both the practical realities and the future directions of digital health education across Europe. The study was conducted as part of the European project “TRANSiTION - Digital Transition and Digital Resilience in Oncology” (project reference: 101101261) cofunded under the EU4HEALTH program [13]. While the focus of the TRANSiTION project is on the cancer workforce, the emphasis of this study was broader, focusing on the available educational opportunities for HCPs and health managers.

### Study Design and Data Collection

The data were collected through an online survey that was developed explicitly for the purpose of this study and followed the CHERRIES (Checklist for Reporting Results of Internet E-Surveys) checklist, which provides recommendations for improving the quality of web surveys [14]. The survey consisted of 23 questions (Table 1), which were categorized into five domains: general information of the program (6 items), reaction (2 items), learning (5 items), behavior (4 items), and results (6 items). The survey was developed in 3 steps, including a scoping review of the literature to extract any relevant tools that were designed to elicit the desired information. As no questionnaire was retrieved, researchers reviewed the existing literature on how to map educational programs and what the competencies for digital skills in health include. A draft set of questions was created, and a panel of experts identified from the project’s consortium reflected on the proposed draft version. The last phase was to formulate the items according to the Kirkpatrick model [15,16]. The Kirkpatrick 4-level training evaluation model is a globally recognized method for evaluating the results of training and learning programs. The items were rated on a Likert scale (0‐1) for the general subscale, where 0 denotes “No” and 1 denotes “Yes,” and on a Likert scale (0‐2) for the other subscales, where 0 denotes “No,” 1 denotes “I am not sure/In some level,” and 2 denotes “Yes.” The questions were then piloted with 2 experts in a parallel way, and consensus was reached in an expert panel discussion. Their insights were instrumental in refining the survey items through multiple review rounds, ensuring that the language was clear, the content relevant, and the scales appropriately aligned with the study’s objectives. This expert-driven validation process not only strengthened the questionnaire’s content validity but also ensured that it accurately captures the diversity of digital skills training programs across Europe

**Table 1.** Scales and items of the online questionnaire.

Scale	Items
Reaction	What are the biggest strengths of the training? What are the biggest weaknesses of the training?
Learning	Do participants acquire the knowledge that the training program focuses on? Do participants acquire the skills that the training program focuses on? Do participants acquire the attitudes that the training program focuses on? Do participants acquire the confidence that the training program focuses on? Do participants acquire the commitment that the training program focuses on?
Behavior	Are participants able to teach/transfer their knowledge to others after the training? Are participants able to teach/transfer their skills to others after the training? Are participants able to teach/transfer their attitudes to others after the training? Are participants aware that they have changed behavior after the training?
Results	Is the training style appropriate? Does the training increase employee retention? Does the training increase employee productivity? Do employees have higher morale after the training? Does the training lead to an improvement in quality of care? Are participants satisfied with the training?
General information	Existence of official training Use of digital skills in practice Mandatory qualification Organization providing the training Duration of the training Type of education

The research team proofread and edited the survey based on feedback received. The survey was distributed via email and hosted on Google Forms for a period of 4 weeks (June-July 2023). The email contained information about the aims of the study, and consent to participate was requested by the experts. Informed consent was obtained through an introductory section on the online survey where participants agreed to terms and understood the purpose, confidentiality, and data security measures.

In addition to the survey, information about the training programs on digital skills identified in the different countries (aim and the modules of the training) was provided in English by the experts during a second period (November-December 2023).

### Data Analysis

Data collected from the survey were analyzed using Kirkpatrick 4-level model, which assesses both formal and informal training methods and rates them against four levels of criteria: reaction, learning, behavior, and results [15,16]. Descriptive statistics were used to summarize and describe the main features of the dataset, providing an overview of the data and helping identify patterns. The data related to program information were synthesized and analyzed using the “Digital Competence Framework for Citizens (DigComp)” by the European Commission [17]. The DigComp provides a common understanding of what digital competence is, and was selected because an appropriate and widely accepted framework of digital competencies for professionals in health was not identified. The framework recommends five categories—information and data literacy, communication and collaboration, digital content creation, safety, and problem solving—that every person should be able to obtain, and 21 competencies that training programs should take into consideration. The status of the countries in our sample was scored according to the training program evaluations by introducing a traffic light approach. More specifically, a category was marked as red (poor) when fewer than half of a category’s competencies were met. Yellow was used when at least half of the competencies in a category were included in the training, and green when more than half or all of a category’s competencies were included in the modules.

### Participants

The survey was conducted among a group of 18 European experts with extensive knowledge and experience of the topic. The experts were professionals working in the fields of digital health, health and cancer care, education, research, or policy positions (Table 2). Eligible participants were required to be recognized as national-level experts in digital health technology. This meant they had comprehensive knowledge and relevant experience in the digital health field within their own country. In addition, they were involved in developing, implementing, or promoting digital health solutions or in shaping national policies and strategies aimed at integrating digital technologies into the health care system to enhance patient care, accessibility, and efficiency. At least one expert representing one of the 14 project partner countries was identified through the project’s research network or was a member of the consortium. Eighteen national experts on digital skills training from Belgium, Bulgaria, Croatia, Cyprus, France, Germany, Greece, Italy, Lithuania, Poland, Portugal, Romania, Spain, and Slovenia participated in the study.

**Table 2.** Experts’ characteristics.

Country	Region	Gender	Expert’s background	Type of organization
Belgium	Flemish	M^a^	Cancer nurse, researcher, and manager	Professional society and hospital
Bulgaria	National	F^b^	Physician, researcher, and academic	Professional and scientific society
Croatia	Osijek-Baranja County	F	Physician, researcher, and academic	University
Cyprus	National	F	Researcher and academic	University
France	Brittany	M	Physician	University
Germany	National	M	Researcher and digital solutions expert	University
Germany	National	M	Physician and researcher	University
Greece	National	M	Researcher, cancer nurse, scientific, and policy advisor	Ministry
Italy	National	M	Physician and researcher in cancer and digital health	Hospital
Lithuania	National	F	Digital solutions expert	Company
Poland	Central (Masovian District)	F	Researcher in cancer and digital health	Hospital
Portugal	Porto	F	Medical oncologist	Governmental organization
Romania	National	F	Physician and researcher in cancer and digital health	Hospital
Romania	National	F	Physician and researcher in cancer and digital health	University
Slovenia	National	F	Nurse, academic, and researcher	University
Spain	Andalusia	F	Researcher in cancer and digital health	University
Spain	Galicia	M	Researcher in cancer and digital health	Governmental organization
Spain	Catalonia	M	Academic and researcher in cancer and digital health	University

a M: male.

b F: female.

### Ethical Considerations

This study adhered to ethical principles for research involving humans [18]. Participants’ confidentiality was secured by anonymizing all data and using identification codes to ensure that participant identities could not be linked to their responses. Participants were informed in advance that participation was voluntary, and they could withdraw at any time without consequence. Data were stored securely, and the results are presented in aggregate format to protect participants’ privacy. Ethical approvals were obtained from the Cyprus Bioethics Committee (ΕΕΒΚ ΕΠ 2023 01.184).

## Results

### Overview

From the analysis of the data, in the 6 out of 12 countries (50%), the data represented the situation at specific region, while in the remaining 6 countries, the information corresponded to the national level. Input at the regional level came from the following countries: Belgium (Flemish region); Croatia (Osijek-Baranja County); France (Brittany region); Poland (Central-Masovian District); Portugal (Region of Porto); and Spain (Regions of Andalusia, Galicia, and Catalonia).

### Current Status and Challenges in Digital Health Usage and Optimization

An official training program was provided in 47.9% (23/48) of cases to HCPs and 29.2% (14/48) to nonclinicians, while in 39.6% (19/48), there was no official training in digital skills for the health care workforce. Specific educational programs in oncology were identified in only 4.2% (2/48) of cases. Experts from Bulgaria and Lithuania reported that, in their countries, a training program in digital skills did not exist.

In 64.6% (31/48) of the training programs, participants obtained a certificate upon the successful completion, but only in 31.3% (4.38/14) of the countries’ experts reported this was a mandatory qualification for work or career progression. The type of education was mostly 68.8% (33/48) formal and provided by academic institutions 56.3% (27/48). The courses were either online asynchronous 52.1% (25/48) or with physical presence 35.4% (17/48), demonstrating a preference for self-directed training with flexible timelines.

The duration of the training programs varied from 10 hours to 4 academic semesters, the frequency of their provision ranged from 9 times a month to once annually, and the number of participants was reported to be between 15 and 50 persons. What was paradoxical from the analysis was that, even though there was a relatively scarce provision of opportunities in digital skills education, in 95.8% (45/47) of the cases, both HCPs and health managers were required to use digital skills in their daily practice.

Experts also reported that the targeted population (HCPs and health managers) did not have the chance to participate in the content development or the methodology or tools for training of the programs. Based on this fact, they believed this explains why the programs were not addressing the trainees’ needs in a comprehensive or satisfactory way, nor did they implement learning approaches appropriate for this population. Moreover, even though an evaluation of the training was provided by the participants (ie, posttraining assessment), according to experts’ views, this did not seem to be considered.

In addition, the lack of a national strategy for education in digital technologies for HCPs or health managers was another reason that the training programs did not meet current needs in health and oncology. Only in Greece, Cyprus, Slovenia, and Poland was the existence of a national strategy or legislation reported to be in place. However, the level of execution of these strategies and legislations was highly diverse in these countries.

### Kirkpatrick Model Analysis

The effectiveness and utility of the trainings, including how people put their learning into practice and how this positively impacts their role and the wider organization, were addressed via Kirkpatrick 4-level training evaluation model.

**Figure 1.**
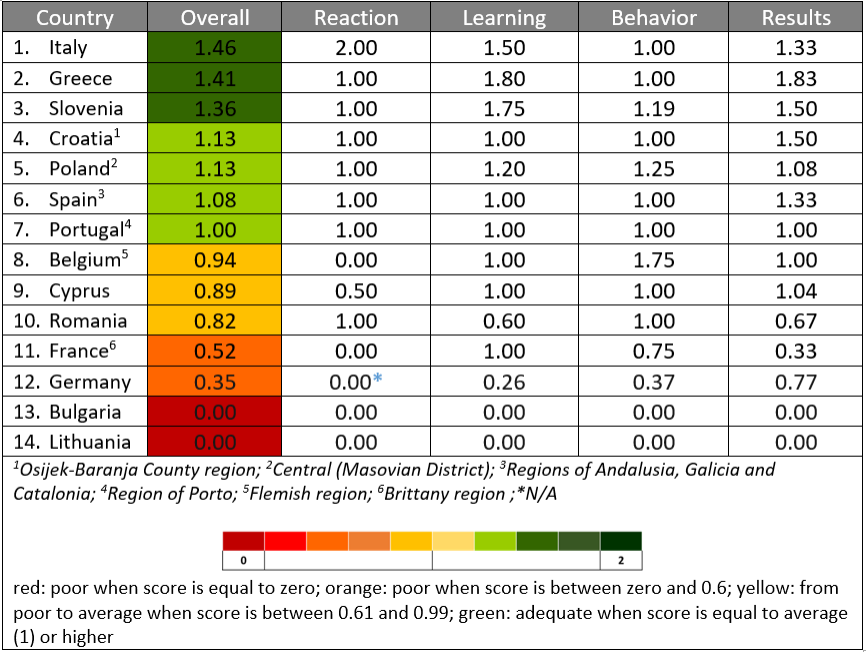
Countries ranking on digital training programs for health care workforce according to Kirkpatrick model scores.

**Figure 2.**
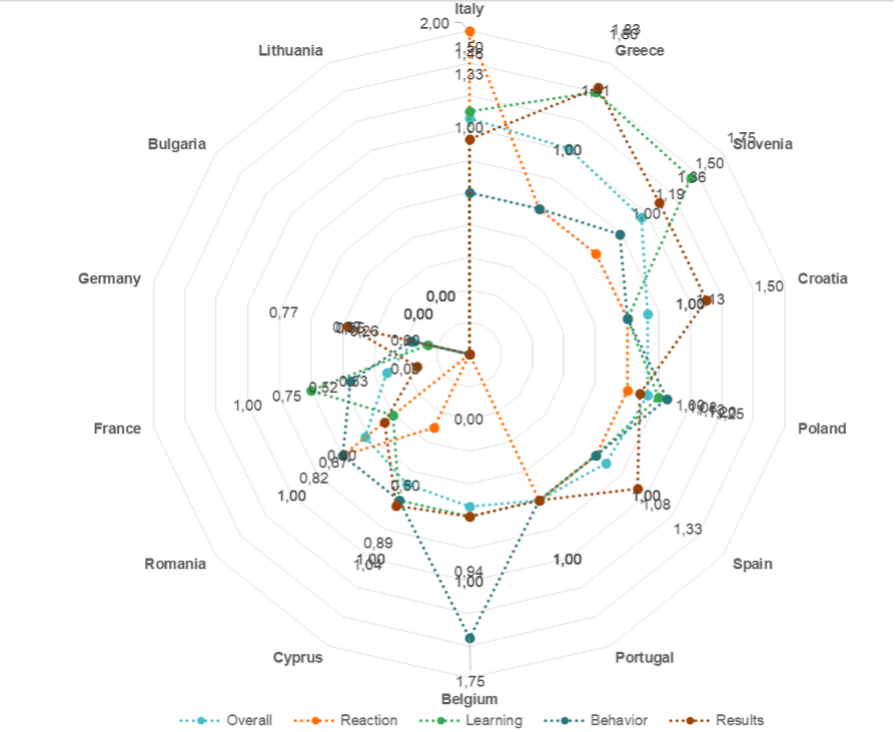
Radar chart of countries scoring on digital training programs for health care workforce according to the Kirkpatrick model (moving from the center, which equals 0, to the last axis that equals 2, better performance is achieved).

The scores of the 4 domains of the Kirkpatrick model, as well as the overall score for the 14 countries, are presented in Figure 1. Countries were ordered according to their overall performance. The color spectrum visualizes the performance status of the countries in the digital skills according to the scores. Moving from red to green, the colors represent better performance. An overall view is also presented in the radar chart (Figure 2). Almost half of the countries scored lower than the mean in overall performance status, which reflects the gap in knowledge and skills of HCPs and non-HCPs.

### Kirkpatrick Model Subscale—Reaction

The satisfaction from the training programs is described from the codes that emerged from the analysis (Textbox 1). Italy scored higher, countries that reached the average score were Greece, Slovenia, Croatia, Poland, Spain, Portugal, and Romania, while Cyprus, Belgium, France, Germany, Bulgaria, and Lithuania had the worst scores.

Textbox 1. **Strengths and weaknesses of the existing training program.**

**Strengths**

- Worthy
- Successful
- Appropriate style of delivery
- Accommodates trainees’ learning styles
- Engaging
- With health applicability

**Weaknesses**

- Lack of experts
- General content (no health applicability)
- Expensive
- Does not address trainees’ needs
- Inappropriate style of delivery
- Lack of interaction and guidance
- Not offered frequently
- Not updated content

According to the experts, the content of the programs did not meet the needs of participants working in the field of cancer care and did not prepare them for current advancements in health technology, which can impact their ability to effectively and efficiently adopt and accept (ie, low acceptability) technological solutions in health.

### Kirkpatrick Model Subscale—Learning

In all items of the learning subscale (ie, acquisition of knowledge, skills, attitude, confidence, and commitment by participants), responses scored more than 60% (35/57) in the option “In some level,” which demonstrates the inability of the trainings to develop participants’ skills, attitudes, and knowledge, as well as their confidence and commitment to digital technologies. Countries scoring higher than the mean were Greece, Slovenia, Italy, and Poland.

### Kirkpatrick Model Subscale—Behavior

Behavior subscale scores were low, less than 10% (5/57), across all items (ie, participants’ ability to teach or transfer knowledge, skills, and attitudes to others and perform behavior change). This means that training graduates were unable to apply and incorporate their training successfully into practice. Countries with scores higher than the mean were only Belgium and Slovenia. This poor outcome reflects the gap between possessing digital skills and effectively imparting these skills to others, due to lack of pedagogical approach and program design, and lack of teaching capacity for enhancing participants’ confidence and role perception.

### Kirkpatrick Model Subscale—Results

The last level of the Kirkpatrick model refers to the results of the training. Participants’ responses indicated negative or neutral outcomes, with scores ranging from 52.1% (29.7/57) to 66.7% (38/57) on the “in some level” and from 10.4% (5.9/57) to 35.4% (20.2/57) on the “no” option for items at this level. This negative outcome supports that existing programs were not addressing participants’ needs in a comprehensive or satisfactory way, nor did they implement ways of learning in an appropriate style for the health workforce. Items included appropriate style of training, increasement of employees’ retention, productivity, morale, satisfaction, and quality of care improvement. Countries that scored higher than the mean were Greece, Slovenia, Croatia, Italy, and Spain.

### Training Programs Competencies Evaluation

**Figure 3.**
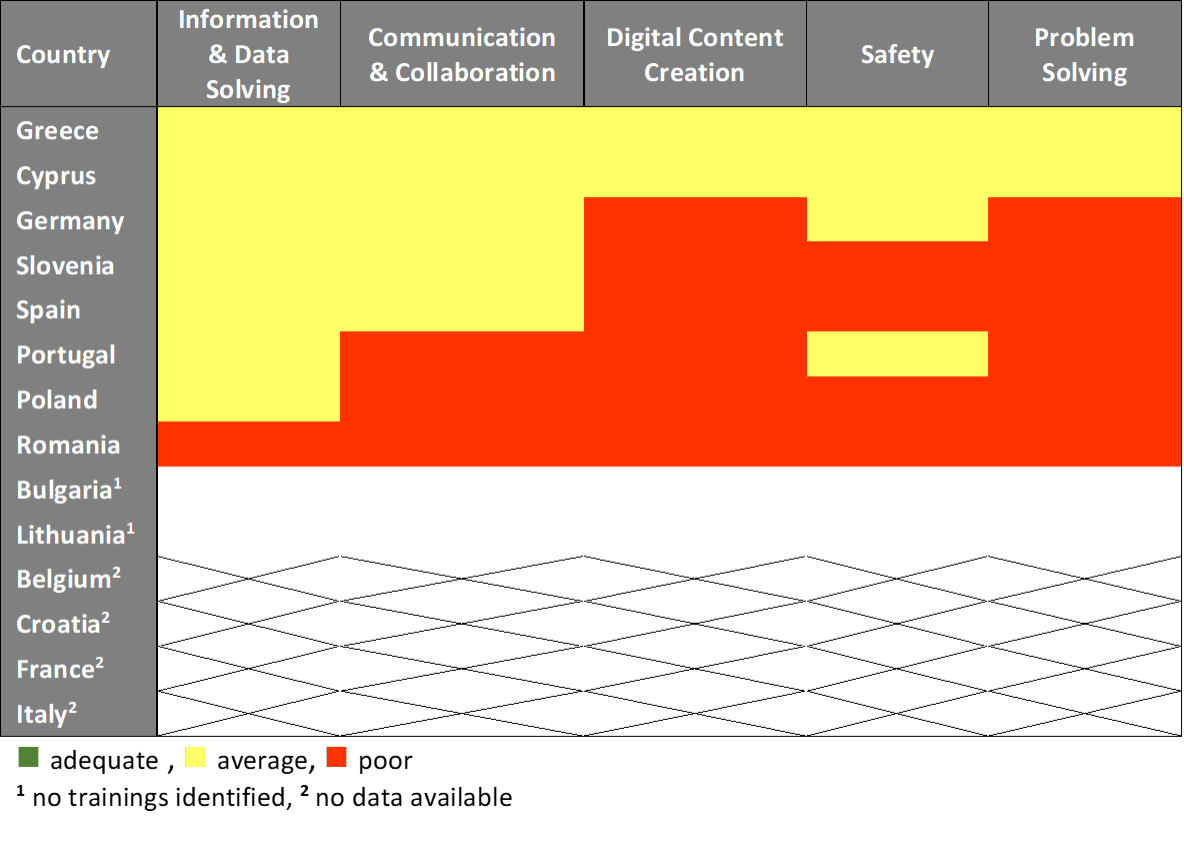
Digital training programs for health care workforce evaluation per country.

The performance of the countries in our sample, assessed by the acquired digital competencies from the educational programs, is presented in a visual snapshot (Figure 3). No country had a sufficient training program in digital skills for the health care workforce, according to the data, as none of the 5 categories was marked as green in any country. As training programs in Bulgaria and Lithuania were not identified, the evaluation of those countries at this stage was left blank. Moreover, there were missing data from 4 of the 14 participating countries, specifically Belgium, Croatia, France, and Italy (response rate 71.43%; 10/14).

The evaluation of the training programs’ competencies identified the weakest areas in digital skills education for HCPs and health managers according to the DigComp framework. Most of the programs lacked adequate skills training for digital content creation and problem solving. The first category enables participants to create and edit digital content, improve and integrate information into an existing body of knowledge, while understanding how copyright and licenses are applied. The second category involves identifying needs and problems, resolving conceptual problems and situations in digital environments, and keeping up to date with digital evolution. The following categories showed average performance in most of the existing programs: safety, communication and collaboration, and information and data solving.

The findings of the evaluation reflect the great need for, and the gaps in, the health workforce’s education in digital skills and health technology application in practice.

## Discussion

### Principal Findings

The aim of this study was to identify and map the existing training programs for HCPs and health managers in digital skills across Europe. Mapping programs across 14 European countries (Belgium, Bulgaria, Croatia, Cyprus, France, Germany, Greece, Italy, Lithuania, Poland, Portugal, Romania, Spain, and Slovenia) highlighted certain patterns, practices, and variations in training methods, strategy, and quality of the programs. The inequalities in access and provision of such educational opportunities, the low quality of the existing programs, the lack of national strategies, and the inability of programs to meet the health care workforce’s needs and current practice requirements demonstrate the variation of digital skills training and complexity of digital skills development across Europe. Countries (Italy, Greece, and Slovenia) with better performance in digital skills training had implemented more extensive strategies, which included specialized training (for medical oncologists, nurses, and health managers), incentives for workforce development (educational leave and career development), and mandatory continuing education (for career progression and job retention).

According to experts’ responses in this study, digital health is increasingly seen as an integral part of the health workforce’s skill set and all roles have some kind of digital element nowadays. At the same time, the gap in digital skills remains high across countries and professional disciplines. Another reason, according to the findings, is the fact that the existing training programs were not addressing the needs of HCPs and the health managers, or they did not link theory, practice, and the unique needs of health care. Therefore, the findings point to the need for the development and provision of comprehensive training in digital skills for professionals in health care settings, within and between countries. This training needs to focus on quality, appropriate design and style, and reflect HCPs and health managers’ preferences and needs. In addition, the accreditation of the program will empower people and will enhance their professional inclusion and employability.

Digital health training programs need to incorporate participants’ aspects, consider their needs, and be adapted to economic, social, cultural, and organizational factors [19-21]. The training programs should address perspectives on various levels (decision-makers, digital health implementers, and users), the profile of trainees (including leadership skills), and highlight the urgent need for monitoring, assessment, and publishing the lessons learned from those experiences [22-25].

Training programs in digital skills represent an important component of future practice, but limited academic and placement opportunities are currently being offered across Europe. Attention should be given to the use of apps in academic teaching and on placement, as well as in delivery of care [26,27]. The lack of training in digital skills in Europe is a significant issue that has been acknowledged by the European Commission as a necessity for the digital transition [28,29]. This acknowledgment has been reflected, for example, as part of the communication from the commission to the European parliament, the council, the European economic and social committee, and the committee of the regions that is encapsulated in the report “2030 Digital Compass: the European way for the Digital Decade” [29]. In the same light, the EU Beating Cancer Plan supports maximizing the potential of digitalization in health care as a means to address inequalities and the provision of patient-centered quality care across the disease pathway [30]. Obstacles to developing successful trainings in the digital area include, among others, the lack of guidelines and competence frameworks, and the low levels of expertise of the training program staff [31-34].

The rapid technology advancements increase the importance of digital skills in the health setting. The digitization of health and the increased reliance on information and communication technology solutions create the demand for professionals with digital skills for career progression and participation in digital care [35-37]. Inequalities in many European countries (ie, both within and between countries) and the lack of the skills required could impact economic growth and innovation. Thus, schools of health sciences need to incorporate digital skills training into their curricula to ensure that students are equipped with the necessary knowledge and abilities. At the same time, it is essential to provide opportunities for continuing education to those professionals already in the workforce to upskill or reskill in digital areas. For that reason, governments, health, and educational institutions should collaborate to provide equal opportunities [38-40].

### Strengths

The findings of this study provide an overview of the current state of digital skills training for the health workforce in Europe, which helps to identify key areas for improvement and can serve as a benchmark for measuring progress over time. It also highlights gaps in knowledge, skills, and resources that are crucial for developing targeted strategies and can inform policy makers and other stakeholders, enabling them to make more informed decisions and allocate resources effectively. The findings could be used to raise awareness about digital skills training and serve as advocacy tools to promote change and improvement in the health digital transformation.

### Limitations

Limitations include, first of all, the lack of depth. Mapping studies provide a broad overview of the existing phenomenon, and particularly this survey did not delve deeply into the content of training programs. This is due to the fact that the main focus of the study was on identifying and categorizing the available evidence, rather than critically appraising or synthesizing it. Nevertheless, aspects of the quality of the training programs were captured by the Kirkpatrick model. This has limited the likely impact on the reliability and validity of the findings and may not provide a complete picture of the available evidence. Moreover, in half of the participating countries, the data did not represent the situation on a national level. Finally, a limitation identified in the study is the retrieval of data from regional areas in specific countries rather than national data. Nevertheless, based on the experts’ views, these regions reflect the national status in these countries and therefore are considered representative.

### Implications for Future Research and Practice

Implications for future research and practice highlight the urgent need for a coordinated approach to enhance digital literacy among HCPs and health managers. The implementation of extensive training programs that will cover specialized training, ensuring they are relevant to the clinical realities, up-to-date with technological advancements, and accessible to all, is recognized as a high priority, with policy support.

Digital competencies are increasingly essential for HCPs and health managers in their daily practice, yet participation in digital training is not mandatory for most. This disconnect creates a significant gap between the skills required on the job and the training provided, ultimately contributing to the suboptimal adoption and integration of digital technologies in health care settings. By not ensuring consistent, required education in digital skills, organizations inadvertently perpetuate barriers to innovation, impeding improvements in patient care, efficiency, and overall service quality.

Moreover, research approaches to monitoring and evaluating the training initiatives, and implementing innovative methods of learning could significantly improve the digital competencies and skills of the health care workforce, ultimately enhancing patient care and health systems’ efficiency.

### Conclusion

The mapping of current opportunities in digital skills education and training for the health care workforce reveals a significant variance in the availability, quality, and effectiveness of programs across Europe. Key findings indicate that none of the surveyed countries demonstrated robust digital training, and notable gaps were identified, particularly in specialized training and continuous professional development. Addressing the lack of digital skills training in health requires a multifaceted approach, involving government policies, educational institutions, businesses, and individuals themselves. By addressing this issue, Europe can better equip its health workforce to thrive in the digital era and maintain its competitiveness on a global scale.

## Abbreviations

CHERRIES: Checklist for Reporting Results of Internet E-Surveys
DigComp: Digital Competence Framework for Citizens
HCP: health care professional
ICT: information and communication technology
